# Authors’ Response to Peer Reviews of “Patient Recommendations for the Content and Design of Electronic Returns of Genetic Test Results: Interview Study Among Patients Who Accessed Their Genetic Test Results via the Internet”

**DOI:** 10.2196/37170

**Published:** 2022-05-31

**Authors:** Diane M Korngiebel, Kathleen McGlone West

**Affiliations:** 1 University of Washington Seattle, WA United States

**Keywords:** user-centered design, genomic medicine, patient portals, electronic health records, return of results, bioethics, EHR, genetics, genetic testing, patient preferences, design, human factors

*This is the authors’ response to peer-review reports for “Patient Recommendations for the Content and Design of Electronic Returns of Genetic Test Results: Interview Study Among Patients Who Accessed Their Genetic Test Results via the Internet."*

## Round 1 Review

### Anonymous [1]

#### Introduction

A1. The actual purpose and study rationale/goal of the study [2] was not described until the middle of the *Methods* section (minus the abstract). At the end of the *Introduction*, no information about the study was provided, and so, I was a little lost when transitioning from the *Introduction* to the *Methods* section for a study that hadn’t been mentioned at all. The second sentence in the *Data Collection* section could be moved up as the last sentence of the *Introduction*.

Response: We have stated the purpose in the *Introduction* and moved the second sentence in *Data Collection* to the last sentence in the *Introduction* and revised it for clarity.

A2. Toward the end of the *Introduction*, the inclusion about barriers to the utilization of patient portals is very broad and not specific to genetics. I would suggest limiting it to genetic test results.

Response: Regarding the suggestion on patient portal use to return genetic test results, the literature does not focus on such a use for patient portals but rather on patient portal use in general. The authors suspect that this may be due in part to the history of all genetic test results being deemed especially fraught and complicated to return by default, and thus many genetics professionals do not support electronic return or only support very limited electronic return.

#### Methods

A3. Perhaps include a *Study Overview* section before *Participant Recruitment* if you do not wish to introduce the study in the *Introduction*.

Response: We have introduced the study in the *Introduction*.

A4. Either provide the semistructured interview guide or provide more detail about the content and structure (eg, funnel approach?).

Response: We have selected sample questions from the semistructured interview protocols and have included them in Table 2.

A5. There is no mention of the analysis of content-related themes in the *Data Analysis* section.

Response: We have added details about our analytic process in the *Data Analysis* section, including the specific direct content analysis approach we applied to identify the details of the design elements.

#### Results

A6. Confirm whether the patient demographics were the same for both study groups. Perhaps redo the table to include a breakdown of demographics between the two groups.

Response: We have reworked the demographics table to depict the study groups separately.

A7. Clarify if the content recommendations came from the group that was asked to compare their experiences receiving genetic vs nongenetic test results through a patient portal.

Response: We can confirm that the quotations presented as exemplars were from the sections of the protocol where genetic test results were being discussed. That being said, participants frequently switched back and forth when discussing genetic and nongenetic test results. That level of fluidity is a finding that will be reported in another paper on thresholds for the electronic return of genetic and other test results.

A8. Did you conduct any analysis to factor in patients’ background (eg, education, gender, age) or the specific type of experience with genetic testing to provide some context of their responses?

Response: We did not do an additional analysis, as the overall topics seemed similar across participants, and as the reviewers have indicated, the study is small and exploratory.

A9. Without a better understanding of what the questions were, it is not totally clear if the questions were totally open-ended or if you asked them to provide feedback on specific suggestions (like the summary sheet). I assume the questions were more open-ended, given the data analysis description, but the results appear to be narrowly confined.

Response: We have included Table 2, which presents sample questions. One of the strengths of a semistructured protocol is that it allows interviewers to organically adjust questions both in real time and when moving forward with future interviews. Many design-related data from participants, such as the suggestion to include a summary, were collected this way.

A10. It seems to me that design recommendation #3 about smartphone functionality is not specific to genetics and should not be reported as a recommendation.

Response: We think it is important to include smartphone functionality, despite the fact that smartphone access to test results would apply to all types of tests available online. It is crucial that report template designers understand that many patient users will be accessing those results on smartphones to, for example, share their genetic variant information with a new medical provider outside of their system.

A11. Some confusion about recommendations—is a simple coversheet (design recommendation #1) the same as an electronic summary (design recommendation #2) and a patient-friendly results summary (domain 2 subheading, content recommendations #2-#4)

Response: The summary is the same as the coversheet. We have changed the language we use to be consistent in conveying this.

#### Discussion

A12. Include some discussion of the implementation of the recommendations. Many would take considerable time to complete for multiple testing vendors/lab reports. Are they really feasible? Do you anticipate that the laboratories will do some of this work or will it fall to test orderer?

Response: Although we understand that there will be challenges regarding the implementation of any templates and processes for the electronic return of genetic (and other) test results, the focus of this study was on gathering patient user feedback and advice. We acknowledge this limitation on page 17: “We acknowledge that a patient-centered approach may elicit suggestions for content and design that might not be easily accommodated by available patient portal software (such as that available through Electronic Health Record software) or by the clinical workflow of healthcare systems or preferences of individual providers. These issues are beyond the scope of our study but must be considered in the final decisions regarding portal-based return of genetic results.”

A13. In the section *Comparison to Prior Work*, I would suggest including more discussion about the format and design of current lab reports. Many are made available through labs on their websites. It is difficult to generalize lab reports for different indications/purposes and come up with a best fit with respect to design/formatting. Certainly, patient feedback will be valuable for learning how to improve the comprehension of genetic testing lab reports. Many results cannot be analyzed without the consideration of more clinical information. Test reports are intended for health providers and thus the style, jargon, and information will understandably differ for patients. The authors should consider reviewing reports intended for patients (eg, 23andMe), which are delivered electronically.

Response: Comparisons of industry methods and content for the return of results would be useful but are beyond this study. That being said, as authors who work in academic settings, we are aware that academic medical centers and health care organizations likely do not have the bandwidth to provide the type of test report and test report technical support that for-profit companies can.

#### Minor Comments

A14. Remove the extra numbers outside at the bottom of table.

Response: The number that follows Table 1 is a footnote relevant to the table. We have replaced the number with an asterisk.

### Anonymous [3]

#### Major Comments

B1. In the final paper, I would recommend not including the quoted comments from the qualitative interviews. I would put those in the supplemental materials, as they are interesting, but they do not add that much to the paper itself.

Response: While we understand that removing quotations is economical, these exemplars are the “figures” of qualitative research that allow others to judge some of our interpretations of the data. We prefer to leave them in the main body of the text rather than move them to a supplementary file, so that readers can more easily judge our work. We have kept the number of our exemplar quotations to a minimum.

#### Minor Comments

B2. One area that is mentioned but not emphasized is the extension of the results of this qualitative study to the communication of nongenetic tests to patients. The same sort of principles should apply in terms of the cover sheet and the detailed explanation. Some of us already do this with our patients, but an extension of this study would allow some evidence to support that practice.

B3. It would be nice to expand the study to include both nongenetic test results and diagnostic imaging results in terms of the design, content, and functionality of the results presentation.

B4. An additional study would be looking at optimizing results presentation and content for smartphones versus computers or tablets. There may be a way to optimize the presentation of the data so that patients could more easily see the data on the smartphone form factor. That is an area for future study.

Response: We cannot actually expand the study at this point, but we agree with the reviewer that there are other relevant areas of application where more research needs to be done.
